# Hybrid Nanodisk Film for Ultra-Narrowband Filtering, Near-Perfect Absorption and Wide Range Sensing

**DOI:** 10.3390/nano9030334

**Published:** 2019-03-02

**Authors:** Wenli Cui, Wei Peng, Li Yu, Xiaolin Luo, Huixuan Gao, Shuwen Chu, Jean-Francois Masson

**Affiliations:** 1 College of Physics and Optoelectronics Engineering, Dalian University of Technology, Dalian 116024, China; xcuiwenli@163.com (W.C.); 1050027560@mail.dlut.edu.cn (L.Y.); shark@mail.dlut.edu.cn (H.G.); chuswdlut@163.com (S.C.); 2 College of Science, North University of China, Taiyuan 030051, China; 3 College of mechatronic Engineering, North University of China, Taiyuan 030051, China; ouwenlxl@163.com; 4 Department of Chemistry, University of Montréal, Montréal, Quebec H3C 3J7, Canada; jf.masson@umontreal.ca

**Keywords:** multifunctional, hexagonal nanodisk film, ultra-narrowband filtering, near-perfect absorption, wide RI sensing

## Abstract

The miniaturization and integration of photonic devices are new requirements in the novel optics field due to the development of photonic information technology. In this paper, we report that a multifunctional layered structure of Au, SiO_2_ and hexagonal nanodisk film is advantageous for ultra-narrowband filtering, near-perfect absorption and sensing in a wide refractive index (RI) region. This hexagonal nanostructure presented two remarkable polarization independent plasmon resonances with near-zero reflectivity and near-perfect absorptivity under normal incidence in the visible and near-infrared spectral ranges. The narrowest full width at half maximum (FWHM) of these resonances was predicted to be excellent at 5 nm. More notably, the double plasmon resonances showed extremely obvious differences in RI responses. For the first plasmon resonance, an evident linear redshift was observed in a wide RI range from 1.00 to 1.40, and a high RI sensitivity of 600 nm/RIU was obtained compared to other plasmonic nanostructures, such as square and honeycomb-like nanostructures. For the second plasmon resonance with excellent FWHM at 946 nm, its wavelength position almost remained unmovable in the case of changing RI surrounding nanodisks in the same regime. Most unusually, its resonant wavelength was insensitive to nearly all structural parameters except the structural period. The underlying physical mechanism was analyzed in detail for double plasmon resonances. This work was significant in developing high-performance integrated optical devices for filtering, absorbing and biomedical sensing.

## 1. Introduction

Surface plasmon polaritons (SPPs) are electromagnetic waves propagating at a metal dielectric interface [1], for which resonance arises in a metallic sub-wavelength structure due to localized SPPs or propagative SPPs. This optical phenomenon has spawned a series of applications in nanophotonics [2], sensing [3], and biophotonics [4], among others. As the optical properties are structure-dependent, a broad range of metal-dielectric nanostructures has been investigated theoretically and experimentally. Different nanostructures can exhibit various optical properties applicable to nanoscale waveguides [5], absorbers [6,7], unidirectional couplers [8,9], plasmonic biosensors [10] and filters [11], etc. For instance, the metallic nanodisk structure is a promising candidate for multiple optical applications. Nanodisks are simple to fabricate with a series of photolithography and colloidal lithography techniques [12]. They have been shown to possess excellent optical properties for sensing [13], filtering [14], and near-perfect absorption [15]. Complex nanostructures with nanodisks embedded in gold film have been proposed to improve sensing [16]. With these types of structures, the full width at half maximum (FWHM) has been measured at 13–14 nm [17]. Layered nanostructures consisting of gold, SiO_2_ and over-coated with either asymmetric elliptical nanodisk arrays or square nanodisk arrays have been proposed for near-perfect absorption in the infrared range [15,18]. Unfortunately, previous reports on these nanostructures have usually intensively discussed one or two of the above-mentioned optical characteristics. The nanostructures with more typical optical properties or performance are usually not mentioned and lack detailed reports.

In this paper, we systematically investigated the use of a layered structure of Au, SiO_2_ and hexagonal array of Au nanodisks, which can perform well in refractive index (RI) sensing, ultra-narrowband filtering and near-perfect absorption simultaneously, compared to other nanostructures such as square and honeycomb-like nanostructures. We first demonstrated its performance as a dual channel narrowband filter in the visible and near-infrared spectral regions. The nanostructure also exhibited near-zero reflectivity and transmissivity for both plasmon resonances, leading to near-perfect absorption. We revealed that the first plasmon resonance was associated with a metal–solution interface and was very sensitive to RI, while the second sharp plasmon resonance was mainly associated with propagative surface plasmons (PSPs) of the inner Au film and an optical magnetic resonance of the dielectric interlayer, thus insensitive to RI. Additionally, the tuning of the structure parameters on the reflection spectra for dual plasmon resonances was also subsequently investigated. This nanostructure can integrate a wide variety of functions into a single device, and can be specifically applied in the development of double-channel narrowband filtering, plasmonic narrow spectral absorbers, chemical sensing and biomedical diagnostics.

## 2. Structure and Characterization

**Figure 1:**
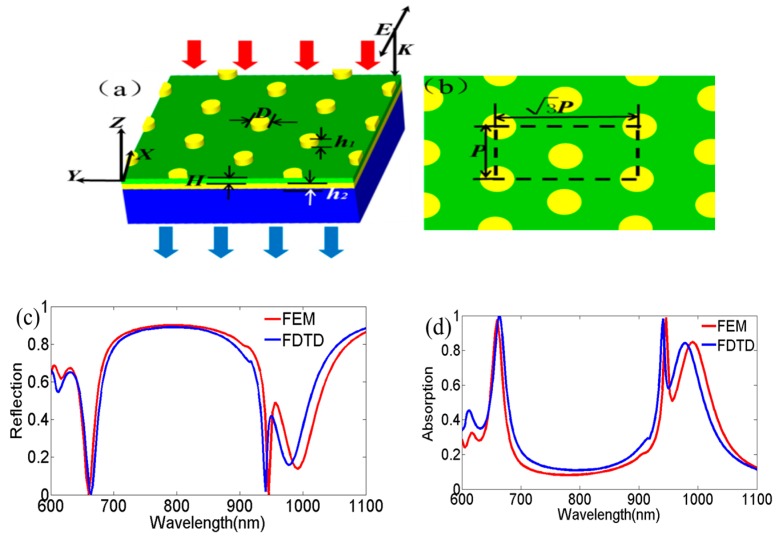

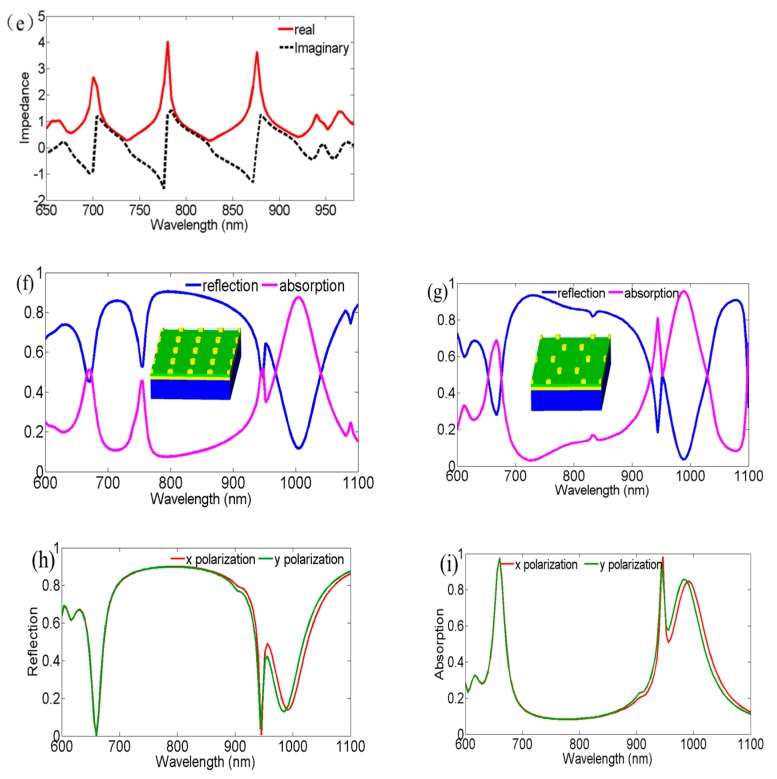
Schematic view and optical properties of the hexagonal nanodisk array layered structure under normal incidence. (**a**) Three-dimensional view of the designed layered structure and its structural parameters; (**b**) top cross-section of the designed structure. Calculated spectra for (**c**) reflection and (**d**) absorption with structure parameters (*P*, *D*, *h*_1_, *h*_2_, *H*) = (700, 200, 50, 50, and 40 nm) by finite element method and finite-difference time domain simulation, respectively; (**e**) real part and imaginary part of the retrieved relative effective impedance in discussed wavelength regions. Calculated reflection (blue line) and absorption spectra (pink line) for (**f**) square nanodisks array and (**g**) honeycomb-like nanodisks array with the same structure parameters as that of discussed hexagonal nanodisks array structure. Insets of (**f**,**g**) show the schematic views of square array and honeycomb-like array, respectively. The influence of changing polarization directions onthe (**h**) reflection and (**i**) absorption spectra.

The optical properties of the layered nanostructure of Au, SiO_2_ and the hexagonal array of Au nanodisks were thoroughly investigated using simulations (see Figure 1). Figure 1a,b illustrate the schematics of the proposed layered structure, where the top layer is an array of gold nanodisks with the height of *h*_1_ = 50 nm and a diameter of *D* = 200 nm. The nanodisk array rested on a SiO_2_ layer of *H* = 40 nm thickness, which also rested on a gold layer of *h*_2_ = 50 nm thickness. The center-to-center distance between two nearest-neighbor nanodisks was *P* = 700 nm. This layered nanostructure was deposited on a BK7 substrate with thickness larger than 100 nm. The layered nanostructure can be fabricated using standard processes [19]. The gold and SiO_2_ films were deposited on a glass substrate by electron-beam (E-beam) evaporation, which was followed by the deposition of a resist (for example: Zep520A), then patterned in the hexagonal nanodisk array with electron beam lithography (EBL). After development, the top layer gold was deposited onto the sample and the remaining resist could be lifted-off to yield the layered nanostructure. The optical properties of this layered structure were investigated using the commercial software COMSOL MULTIPHYSICS (5.2 Version, Comsol company, Stockholm, Sweden), which is based on the finite element method (FEM). Here, the wave optics module and frequency domain interface were employed in order to solve for time-harmonic electromagnetic field distributions. The permittivity of Au was described using the Drude–Lorentz model [20], which is given by:

(1) $$\varepsilon_{m}\left(\omega \right)=\varepsilon_{r}-\frac{\omega_{p_{0}}^{2}}{\omega (\omega +i\gamma_{0})}-\frac{\Delta \varepsilon_{0}\Omega_{0}^{2}}{\omega^{2}-\Omega_{0}^{2}+i\omega \Gamma_{0}}$$

In Equation (1), the first two items are given by the Drude model, where ω is the angle frequency, $\omega_{p_{0}}$ is the plasma frequency, and $\gamma_{{}_{0}}$ is the damping coefficient. The third term is the Lorentzian term, where $\Omega_{{}_{0}}$ and $\Gamma_{{}_{0}}$ stand for, respectively, the oscillator strength and spectral width of the Lorentz oscillators and $\Delta \varepsilon_{0}$ can be interpreted as a weighting factor. We used the values of the Drude–Lorentz model as follows: $\varepsilon_{r}=5.9$, $\omega_{p_{0}}=1.33\times 10^{16}$ rad/s, $\gamma_{0}=9.87\times 10^{13}$ rad/s, $\Omega_{0}=4.07\times 10^{15}$ rad/s, $\Delta \varepsilon_{0}=1.09$, $\Gamma_{0}=6.58\times 10^{14}$ rad/s. Silicon dioxide was modeled as a lossless material with the RI *n*_SiO_2__ = 1.45. The RI of BK7 glass was 1.513. The above-mentioned parameters and Equation (1) were set in global definitions of the model builder for using the software. This nanostructure was illuminated in the negative *z* direction by a plane wave with the polarization direction as indicated in Figure 1a. In our simulation, to achieve high precision, improve calculation efficiency and save computer memory, a single unit cell of structure was chosen, as indicated in Figure 1b. The periodic boundary condition was set in the *x* and *y* directions, while perfectly matched layers (PMLs) were used in the ±*z* directions. Moreover, the wave impedances of the PMLs for two directions completely matched the wave impedances of the adjacent dielectrics for air or BK7 in order to damp propagating waves, and absorb transmission and reflection. In order to generate finer meshes for the whole model, we further refined the meshes of the nanodisks region by using a custom function for a high solving precision. The maximum element size was set to 60 nm, and the minimum element size was set to 10 nm, which are both much smaller than the Au nanodisk diameter. The characteristic spectrum was achieved by numerical calculation with a 2 nm resolution in the wavelength range from 600 to 1100 nm. Good convergence was achieved in our simulation calculation. The validity of the results from the FEM method was further confirmed using the finite difference time domain (FDTD) algorithm (Lumerical FDTD Solutions, Inc., Vancouver, Canada) [21], as shown in Figure 1c,d. All simulation results were normalized to the incident light power.

## 3. Results and Discussion

The reflection and absorption spectra were simulated for the layered nanostructure of Au, SiO_2_ and the hexagonal array of Au nanodisks under normal incidence, as seen in Figure 1c,d. Here, the calculated absorptivity, *A*, was obtained by *A* = 1 − *T* − *R*, where *T* and *R* were transmittivity and reflectivity, respectively. We observed that the result of the FEM calculation generally agreed well with the FDTD calculation. Two narrow plasmon resonances were observed at 660 and 946 nm under normal incidence with near-zero reflectivity, as in Figure 1c. The FWHM of the dual plasmon resonances was notably small at 14 and 5 nm, respectively, for 660 and 946 nm. Figure 1d shows that the calculated absorption spectra exhibited near-perfect absorption at both resonances, and were well matched with the impedance matching theory [22] at the dual resonant peaks. As expressed in Figure 1e, the real part of the impedance at both absorption peaks were close to one and the imaginary parts were nearly zero. In comparison, the simulated spectra of square and honeycomb-like structures with consistent structural parameters for hexagonal structures were also presented (see Figure 1f,g). The results showed that the layered nanostructure of Au, SiO_2_ and hexagonal array of Au nanodisks had sharper filtering and higher absorption characteristics. Moreover, in order to evaluate the sensibility in the polarization direction of the incident light of our designed nanostructure, we also calculated the reflection and absorption for the *y* polarization direction in Figure 1a. The simulation results indicated that the optical properties of this hexagonal nanostructure were polarization-independent of the direction of the incident light, as shown in Figure 1h,i. 

**Figure 2:**
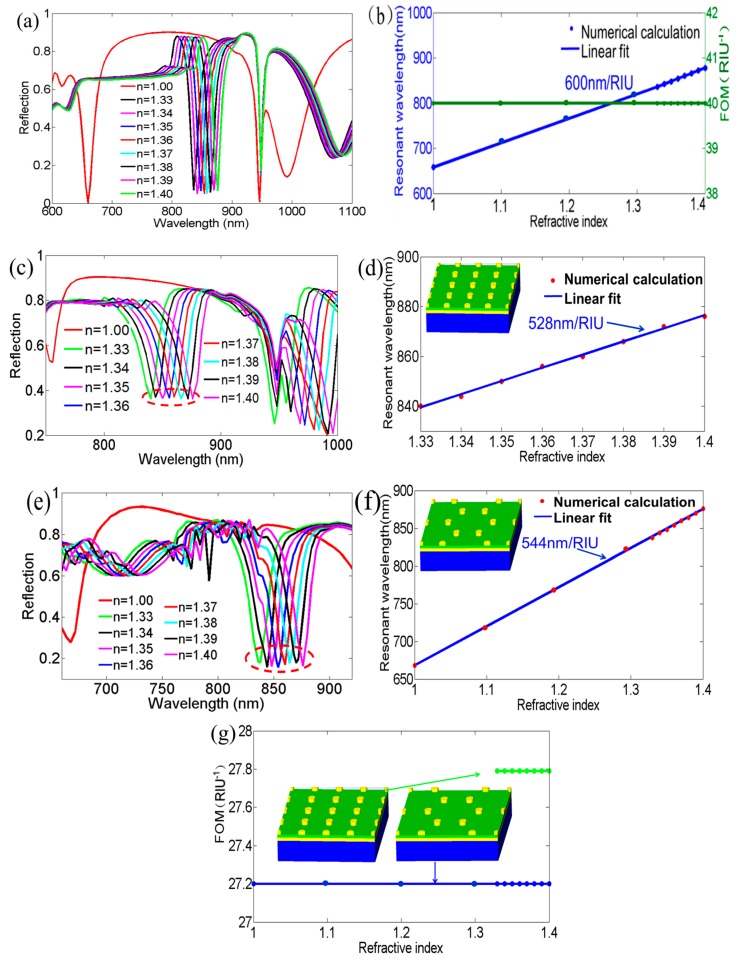
Investigation of the sensing performance of the discussed structure. (**a**) Reflection spectra of the layered structure with different refractive indexes (RIs); (**b**) linear response of the resonant wavelength (blue line) and the figure of merit (green line) at the first plasmon resonance for the designed layered structure. Reflection spectra corresponding to (**c**) the square nanostructure and (**e**) the honeycomb-like nanostructure at various RIs. Responses of resonant wavelengths on different RIs for (**d**) the square nanodisk array and (**f**) the honeycomb-like nanodisk array; (**g**) comparison of the figure of merits with various RIs for the square nanodisk array and the honeycomb-like nanodisk array.

The sensing performance around nanodisks was then simulated in the wide RI range from 1.00 to 1.40 in Figure 2a,b. The first plasmon resonance (at 660 nm in air) showed a linear relationship and a high RI sensitivity (*S* = Δ*λ/*Δ*n*) of 600 nm/RIU (blue line). The figure of merit (*FOM* = *S*/*FWHM*) was also excellent at 40 RIU^−1^ (green line). Coincidentally, the results of the evaluating sensing performance were close to that of the gold-coated nanodisk arrays in our previous reported research [17]. For comparison, the RI responses of square and honeycomb-like structures were also calculated and presented in Figure 2c–f. The computational results indicated that the square array did not present the sensing performance of the whole RI region from 1.0 to 1.4 (data all not shown), therefore, we only provided the linear response of the resonant wavelength in the RI range of 1.33~1.4 in Figure 2d. The honeycomb-like nanodisk arrays demonstrated a linear response in this wide RI region, similar to the hexagonal nanodisk arrays, however, its RI sensitivity was relatively low (see Figure 2f), especially considering the FOM, as shown in Figure 2g. 

**Figure 3:**
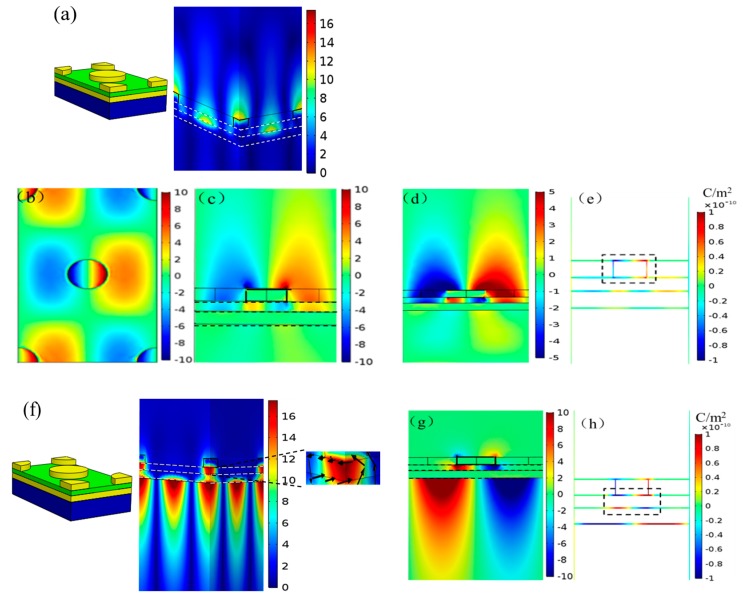
Electromagnetic field and charge distributions for both plasmon resonances with normal incidence. (**a**) Normalized magnetic field distribution at 660 nm; (**b**) normalized electric field distribution for the *z *component (the most dominant component) at the top surface of the nanodisks at 660 nm; (**c**) the electric field distribution for the z component in the cross section through the center of the hexagonal nanodisk array for a unit cell at 660 nm; (**d**) the *z* component of the electric field distributions taken as a *z*-axis slice through the center of the hexagonal nanodisk arrays with a shrinking-scale bar at 660 nm; (**e**) the corresponding charge distribution at 660 nm; (**f**) normalized magnetic field distribution and its partially enlarged distribution at 946 nm. The black arrows represent the direction and intensity of the electric displacement; (**g**) the *z* component of the electric field distribution taken as a *z*-axis slice through the center of hexagonal nanodisk array at 946 nm; (**h**) the correspond charge distribution at 946 nm.

To further investigate the optical properties of the layered nanostructure of Au, SiO_2_ and hexagonal arrays of Au nanodisks, we performed numerical calculations of the spatial distribution of the electromagnetic fields for both plasmon resonances, as shown in Figure 3. For the first plasmon resonance at 660 nm, we observed that the magnetic field was mainly located on the top metal/air interface of the nanodisks and the lower metal film/SiO_2_ interface, as in Figure 3a. The strong localized magnetic field on the top surface of the nanodisks was due to the excitation of the localized surface plasmon (LSP) resonance mode, confirmed from the data presented in Figure 3b,c. The high energy localized on the edge of the nanodisk indicated the stimulation of the LSP resonance mode. Meanwhile, the strong localized electric field on the upper surface of the metal film or the lower surface of SiO_2_ can be understood with the excitation of the SPP resonance mode. The shrinking-scale color bar in Figure 3d exposes this characteristic more clearly. Moreover, we can also distinctly observe that the excitation intensity of the SPP on the Au disk/SiO_2_ surface was stronger than that of the Au film/SiO_2_ surface, in contrast to Figure 3c. Figure 3e shows a charge distribution at 660 nm in the cross-section through the center of the hexagonal array. Expressed by a black-dashed dotted frame in Figure 3e, the obvious oscillating negative charges and positive charges appeared separately on the left and right end faces, corresponding to an electric dipole resonance. Meanwhile, oscillating charge distributions of the Au disk/SiO_2_ surface and the Au film/SiO_2_ surface also appeared. This was compatible with Figure 3c,d. Based on these results, we drew the conclusion that the emergence of a sharp 660 nm is closely associated with plasmonic resonance coupling between the PSP and LSP modes.

We then focused on the second plasmon resonance at 946 nm. The strong magnetic field was mainly confined to the Au film/BK7 substrate interface and the SiO_2_ thin interlayer (Figure 3f). Here, an artificial magnetic moment in the SiO_2_ dielectric spacer layer was strongly induced and resulted in the excitation of the optical magnetic resonance. Additionally, an electric dipole resonance mode was also observed around the nanodisks due to the excitation of the LSP resonance, as revealed in Figure 3g. The charge distribution from Figure 3h more clearly demonstrates this point. The left and right end faces of the nanodisks had distinct negative and positive charges, respectively, which were related to the excitation of the electric dipole resonance mode. Furthermore, the surface charge distribution in the black-dashed dotted frame from Figure 3h revealed the formation of the magnetic dipole resonance mode and the occurrence of the optical magnetic resonance phenomenon. Moreover, the oscillating charges on the Au film/BK7 substrate interface in Figure 3h corresponded to the PSP resonance mode in Figure 3g. Here, for the hexagonal nanodisk array, the excitation of the PSP at the Au film/BK7 interface satisfied the condition below [23]:

(2) $$\frac{2\pi }{\lambda }n_{s}\sin\theta -\frac{2\pi }{P}\sqrt{\frac{4}{3}(i^{2}+ij+j^{2})}=-\frac{2\pi }{\lambda }\sqrt{\frac{\varepsilon_{m}(\omega )\varepsilon_{d}}{\varepsilon_{m}(\omega )+\varepsilon_{d}}}$$

where *P* is the array period, $\varepsilon_{m}$ and $\varepsilon_{d}$ represent permittivity of metal and dielectric, and *i* and *j* are integers. According to Equation (2), we may calculate the theoretical wavelength for the (1,0) PSP mode, which was 947 nm, which agreed well with the numerical wavelength of 946 nm. On the basis of the above analysis, we drew the conclusion that the physical origin of the second plasmon resonance should be attributed to the three coupling modes, namely optical magnetic resonance, PSP resonance and LSP resonance. Compared to the dual-mode coupled resonances of PSP resonance and LSP resonance at 660 nm in Figure 1c, the multi-mode hybridization seemed to be more beneficial to the narrow spectral width and enhanced electromagnetic field to 946 nm. Considering that the strong localized magnetic field was mostly confined to the dielectric interlayer and Au film/BK7 interface, the plasmon resonance of 946 nm was extremely insensitive to the change of bulk RI.

**Figure 4:**
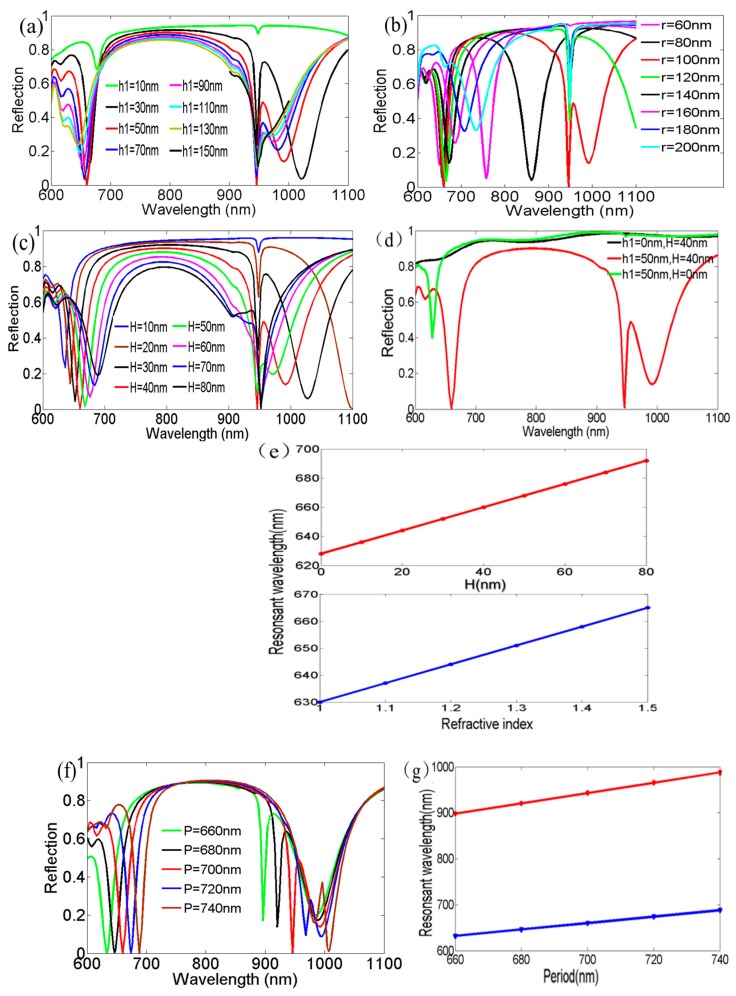
Tuning effects of different parameters on the reflection spectra for the hexagonal nanodisk array at normal incidence. The influence of (**a**) Au disk thickness (*h*_1_); (**b**) Au disk radius *r*; (**c**) SiO_2_ interlayer thickness (*H*) and (**f**) period (*P*) at both plasmon resonances for the calculated reflection spectra; (**d**) comparison of the reflection spectra for different parameters (*h*_1_) and (*H*). The responses of the resonant wavelength at the first plasmon resonance for (**e**) different SiO_2_ interlayer thickness (*H*) and SiO_2_ interlayer RI and (**g**) period (*P*).

Furthermore, we investigated the influence of the structure parameters on the reflection spectra. The thickness (*h*_1_) of the Au nanodisks was increased from 10 to 150 nm in 20 nm steps in Figure 4a. The first plasmon resonance showed a blue shift, which was seemingly an abnormal shifting behavior compared to the usual metal–dielectric–metal-layered nanostructure. We speculated that the phase shift of the incident light on the hexagonal nanodisk array affected the position of the plasmon resonance when (*h*_1_) increased [24]. In addition, *h*_1_ = 50 nm was an optimal value to achieve minimum reflectivity and maximum absorptivity. At this point, the coupling of the LSP excited at the Au nanodisks, the SPPs excited at the Au nanodisk/air interface, and the Au film/SiO_2_ interface became the strongest, corresponding to largest light confinement.

Simultaneously, we also noticed that another plasmon resonance located at 616 nm for *h*_1_ = 50 nm gradually disappeared with the larger (*h*_1_). Concomitantly, the depth of the first plasmon resonance decreased and the FWHM widened due to the merger of the nearby plasmon resonance. Hence, increasing the nanodisk height directly increased the structural radiation damping losses. The second plasmon resonance at 946 nm was constant with the disk height, but the reflected intensity varied with the thickness of the nanodisk, as in Figure 4a. The coupling intensity of the three resonant modes was optimal at a height of 50 nm, resulting in the lowest reflectivity and the largest absorptivity. Furthermore, we also found that the resonance at 992 nm for *h*_1_ = 50 nm became gradually closer to the resonance around 946 nm when (*h*_1_) increased, and eventually disappeared. Meanwhile, the FWHM of the second plasmon resonance widened. This meant that radiation damping losses at 946 nm also increased, similarly to those at 660 nm.

We then examined the impact of the nanodisk radius (*r*) on the optical properties. Increasing the radius from 60 to 200 nm resulted in a redshift of the first plasmon resonance, as seen in Figure 4b. This is attributed to the increased coupling effect between the nanodisks. Meanwhile, the LSP resonant mode excited at the Au nanodisk and the SPP resonant mode excited at the Au film/SiO_2_ interface also shifted, giving rise to the variation of the depth for the first plasmon resonance. The resonance at 946 nm remained constant due to the marginal effect of the radius of the nanodisk on the coupling conditions as seen with Equation (2).

Figure 4c shows the influence of thickness (*H*) of the SiO_2_ dielectric layer on the reflection spectra. There was an obvious redshift with the increasing thickness from 10 nm to 80 nm at the first plasmon resonance, which was opposite to the previously reported results for metal–dielectric–metal nanostructures when changing the interlayer thickness [25]. This opposite shift implies differences in the excitation mechanism between the discussed structure and the other nanostructures. To further reinforce the origin of the first plasmon resonance at 660 nm, we modelled the optical response of a film devoid of nanodisks. The absence of this plasmon resonance was clearly seen for *h*_1_ = 0 nm and *H* = 40 nm (see Figure 4d). Concurrently, we also found that the discussed first plasmon resonance presented for *h*_1_ = 50 nm and *H* = 0 nm, that is, in the absence of the SiO_2_ layer. This result indicates that the thickness (*H*) of the SiO_2_ interlayer did not play a key role in the excitation of the resonance at 660 nm, but mainly served the modulation of the reflective intensity and resonant wavelength position. This is further exemplified in Figure 4e. Increasing (*H*) from 0 to 80 nm led to a linear relationship (red line) between (*H*) and the resonant wavelength at the first plasmon resonance. The RI of the dielectric layer between the Au disks and the Au film was modulated from 1.0 to 1.5. A similar linear relationship (blue line) can be also observed between the RI and the resonant wavelength at the first plasmon resonance. This proves that increasing the thickness of the dielectric layer is equivalent to increasing the effective RI of this same dielectric layer, giving rise to the redshift of the first resonance wavelength. As expected, the resonance at 946 nm was nearly unaffected by the thickness of the dielectric layer for the range from *H* = 10~50 nm. Only when the thickness was above 50 nm was a slight wavelength shift of the second plasmon resonance found, as shown in Figure 4c. This was due to the merger of another nearby plasmon resonance and the second plasmon resonance when (*H*) increased to 60 nm.

Finally, we investigated the influence of the period (*P*) on the optical properties of the hexagonal nanodisk array (see Figure 4f–g). All other parameters were set as shown in Figure 1a,b. With the period increasing from 660 to 740 nm, the simulations showed a redshift in both resonances on the reflection spectra. This agreed well with Equation (2) under the normal incidence for this layered nanostructure of Au, SiO_2_ and hexagonal array of Au nanodisks. In this case, a linear relationship was observed between the periodicity and resonant wavelength, as seen in Figure 4g. This means that we could obtain a certain central wavelength for the filtering function by modulation of the structural period.

## 4. Conclusions

In summary, we numerically demonstrated that a layered nanostructure of Au, SiO_2_ and a hexagonal array of Au nanodisks simultaneously had the properties of extremely narrowband filtering, near-perfect absorption and highly sensitive sensing. We also predicted that this structure would generate two narrowband dips with near-zero reflectivity and near-perfect absorptivity. Moreover, the narrowest FWHM for the dual dips was up to 5 nm, which far exceeds that of others, such as square and honeycomb-like nanostructures. The plasmon resonance at 660 nm was mainly associated with a metal-solution LSP/SPP mode, presenting a high RI sensitivity of 600 nm/RIU and a high FOM of 40RIU^−1^ in a wide RI range from 1.00 to 1.40. The second plasmon resonance was mainly associated with the metal–glass interface and SiO_2_ interlayer, and had great filtering and near-perfect absorption in the near-infrared region. Our simulations clearly reveal that the designed hexagonal nanostructure can be a function of the nanodisk height, radius, thickness of SiO_2_ film and period. Specifically, the tunability characteristic of the filter for a certain central frequency can be well realized by changing the structural period. This work has great potential applications in the development of new miniaturized integration photonic devices such as multichannel ultra-narrowband filtering, perfect absorption and wide RI range biomedical sensing.
